# Long-Lost Relative Claims Orphan Gene: *oskar* in a Wasp

**DOI:** 10.1371/journal.pgen.1002045

**Published:** 2011-04-28

**Authors:** Cassandra G. Extavour

**Affiliations:** Department of Organismic and Evolutionary Biology, Harvard University, Cambridge, Massachusetts, United States of America; University of California Davis, United States of America

## Introduction

“*To us as embyrologists and men the formation of an embryo has appeared to be everything, the history of the germ cells a secondary item of no particular moment. Nature, on the other hand, reverses the relative importance of the two, setting the germ-cells in the place of honour, as linking the remote past with the distant future*.”

In 1902, the vertebrate embryologist James Beard wrote these words in his monograph on germ cells in the skate *Raja batis* [1]. Over 110 years later, germ line specification and development are indeed major areas of investigation in the fields of developmental biology and evolution. August Weismann's description of the germ line as containing “unalterable accessory idioplasm” [2] may sound suspiciously mythical to modern readers. Nevertheless, we now know that in some animals, a special cytoplasm containing conserved gene products is indeed transmitted from oocyte to embryonic germ cells, and again to oocytes in the next generation. This “germ plasm” is necessary and sufficient for germ cell formation, and its molecular basis is best understood in the fruit fly *Drosophila melanogaster*. Germ plasm in some form has been described in oocytes and embryos of most “higher insects” (Holometabola: e.g., flies, wasps, and butterflies) as well as in other animals such as frogs and fish. However, in “lower insects” (Hemimetabola: e.g., grasshoppers, mayflies, and cockroaches) and in most other animals, nothing like germ plasm or inherited germ line determinants have been reported. In mice, the best studied example of such cases, inductive signals from specific somatic cells cause neighbouring cells to adopt germ cell fate. Comparative analyses suggest that most animals may use inductive signaling rather than germ plasm to specify germ cells, including animals branching close to the base of the animal tree (e.g., sponges and cnidarians). This has led to the hypothesis that the ancestral mechanism for animal germ cell specification may have been based on inductive signaling, meaning that germ plasm-driven mechanisms would have evolved independently several times in animal radiation [3]. How such a novel mechanism could have evolved remains unclear. In a recent paper [4], Jeremy Lynch and colleagues provide evidence that a critical component of germ plasm in insects is more ancient than previously thought, and that the driving force for this novel mechanism was the evolution of a novel gene.

## The Lone Ranger

Insects fall into two major groups: the Holometabola (“higher insects”) show indirect development through a pupa or chrysalis stage, while the Hemimetabola (“lower insects”) develop directly, without metamorphosis. All orders of holometabolous insects contain species where, as observed for *Drosophila*, germ cells are exclusively derived from a small number of cells that form at the posterior pole of the embryo shortly after fertilization [5]. These “pole cells” have also been described in some beetles and hymenopterans (bees, ants, and wasps), including the wasp *Nasonia vitripennis*. However, other members of the same insect orders, including the beetle *Tribolium castaneum* and the honeybee *Apis mellifera*, do not form pole cells [6], [7], and the molecular mechanism used to specify germ cells in these insects is presumed to be inductive. Similarly, hemimetabolous insects such as cockroaches and grasshoppers do not have pole cells [8], [9].

Pole cells acquire their germ cell fate by inheriting cytoplasmic determinants, or germ plasm. In *Drosophila*, when germ plasm is removed or destroyed, pole cells cannot form and the animal is sterile [10], [11]. Conversely, transplanting germ plasm to ectopic locations causes ectopic germ cells to form [12], [13]. It turns out that there is only one gene described in *D. melanogaster* whose products are also necessary and sufficient for germ cell formation: *oskar*. Uncovered in genetic screens for maternal effect mutations [14], its transcript and protein are localized to the posterior cytoplasm of the oocyte and early embryo. When overexpressed in ectopic locations, *oskar* induces ectopic germ plasm and germ cell formation [15], [16].

Surprisingly, unlike many other genes with indispensable roles in development, *oskar* is not a widely conserved gene: it proved absent from the first non-fly insect genomes sequenced, and has no clear homologue in any other animal. Although the orthologue from another fly (*Drosophila immigrans*) can substitute functionally for *D. melanogaster osk* [17], that from an equally distantly related fly (*Drosophila virilis*) cannot [18]. This suggests that the fruit fly strategy for assembling germ plasm evolved very recently, in the lineage leading to the Diptera (flies and mosquitoes), but is not widely applicable in other insects. Because *oskar* encodes for a novel protein with unknown function, its evolutionary origins have remained an even deeper mystery. Lynch and colleagues now pull back the curtain on the evolution of *oskar*, revealing that it evolved in higher insects long before the appearance of fruit flies.

## The Search for Family

The wasp *Nasonia vitripennis* belongs to the Hymenoptera (ants, bees, and wasps), which are likely to be the most basally branching order of holometabolous insects [19]. *Nasonia* is an attractive model to study the evolution of germ plasm, because it is easy to culture in the lab, has a sequenced genome, robust protocols for gene expression and functional analysis, and derives its germ line from pole cells. Examining the sequenced genome of this wasp, Lynch and colleagues found an *oskar* orthologue (*Nv-osk*) using a relaxed and modified BLAST strategy. They found that part of the protein has similarities to a family of proteins called tudor-domain-containing (Tdrd) proteins, some of whose members have documented roles in germ cell development in other animals. This suggests that *oskar* may have evolved by duplication and subsequent divergence of a gene that already had a germ cell role. As in *Drosophila*, *Nv-osk* is localized to the posterior of the oocyte and early embryos, and knocking down *Nv-osk* by RNAi results in disrupted germ plasm and no pole cells. However, it also results in a range of somatic patterning defects, suggesting that unlike fly *oskar*, *Nv-osk* may play complex roles outside of the germline as well. The authors then investigated the upstream regulation of *Nv-osk* by examining the roles of two genes that regulate *oskar* translation in flies, *bruno* and *Hrp48*. Knockdown of the wasp homologues of these translational regulators resulted in abnormally localized *Nv-osk* transcripts, suggesting that some aspects of *oskar* regulation may also have ancient roots.

*Nasonia*'s phylogenetic position means it is possible that any characters that it shares with *Drosophila*, including *oskar*, were present in the last common ancestor of all holometabolous insects. However, several holometabolous insects lack pole cells, including *Nasonia*'s close relative the honeybee (*Apis mellifera*), whose genome also lacks an *oskar* homologue. This suggests that *oskar* or germ plasm may have been secondarily lost in some higher insect lineages. To determine whether the *oskar*/germ plasm/pole cells relationship was conserved in other hymenopterans, Lynch and colleagues searched for, and found, an *oskar* homologue in the ant *Messor pergandei*. *Mp*-*osk* transcripts localize to the posterior of oocytes and embryos, and the embryos of these ants have pole cells.

## Back to Our Roots

The authors' choice of model organism and use of multiple dipteran *oskar* orthologues as queries to their wasp genome allowed them to find an *oskar* homologue in a lineage further removed from *Drosophila* than had been previously suspected. This work has not simply added another sequence to our meager list of *oskar* homologues; it also predicts that the origins of this gene could be at least 300 million years old (the estimated time of divergence of Hymenoptera from Diptera [20]). A further prediction from this work is that higher insects as diverse as beetles, moths, and fleas should have *oskar* homologues as well. Given the apparent rapid evolutionary rate of this gene and the absence of genome sequences for most of these insects, these homologues may be challenging to identify, but their study could yield further important insights into the evolution of germ line specification in these animals.

What about other animals, like *Xenopus*, *Caenorhabditis elegans*, and zebrafish, which have maternally inherited germ line determinants but no *oskar* homologues? A zebrafish gene called *bucky ball* has been reported to have *oskar*-like genetic properties, but has no detectable homology to *oskar* [21]. However, the work on *Nv-osk* sheds light on this problem as well. We know that the genetic networks regulating germ cell development on the one hand, and subcellular localization mechanisms including translational control on the other hand, are ancient metazoan mechanisms [22], [23]. This suggests that the advent of novel *oskar*-like molecules capable of interacting with both networks could have facilitated the evolution of novel modes of specifying germ cells. Future work could take advantage of this prediction based on known modularity of mechanisms, by searching for germ plasm nucleators on the basis of molecular properties, rather than traditional homology.

## Future Generations

Finally, finding more *oskar* homologues may give us insight into mechanisms of neofunctionalization and the evolution of novel protein functions. While one region of *oskar* may have its origin in a duplicated Tdrd gene, the C terminus of Oskar has the greatest (but still weak) similarity not to animal gene domains, but to SGNH/GDSL hydrolases of bacterial species! Lynch and colleagues discuss the possibility that horizontal gene transfer from bacterial endosymbionts could have led to the fusion of domains from animal and insect genes. Although speculative, this is not completely outside the realm of possibility, as there is a widespread association between insects and endosymbiotic bacteria, which have often been found to colonize the germline of their hosts.

Understanding the evolutionary processes that created this puzzling gene will undoubtedly be more difficult than elucidating its mechanism of action in extant animals. Nevertheless, the effort will be worth the reward, as thinking broadly about the origins of genetic innovation can help us understand not just how new genes can arise, but also how these new genes can lead to the evolution of novel developmental mechanisms.
